# Changes Related to Age in Natural and Acquired Systemic Self-IgG Responses in Malaria

**DOI:** 10.1155/2011/462767

**Published:** 2011-12-29

**Authors:** Romuald Dassé, Didier Lefranc, Sylvain Dubucquoi, Patricia Dussart, Virginie Dutoit-Lefevre, Boualem Sendid, François Sombo Mambo, Patrick Vermersch, Lionel Prin

**Affiliations:** ^1^Laboratoire d'Immunologie EA 2686, IMPRT-IFR 114, Faculté de Médecine Pôle Recherche, Université Lille 2, 1 Place de Verdun, 59045 Lille Cedex, France; ^2^Laboratoire d'Immunologie et Hématologie du CHU-Cocody, Abidjan, Cote D'Ivoire; ^3^Laboratoire de Parasitologie et de Mycologie, Institute de Biologie et Pathologie, CHRU de Lille 59037 Lille, France; ^4^Service de Neurologie D, Hôpital Roger Salengro, 59037 Lille Cedex, France

## Abstract

*Background*. Absence of acquired protective immunity in endemic areas children leads to higher susceptibility to severe malaria. To investigate the involvement of regulatory process related to self-reactivity, we evaluated potent changes in auto-antibody reactivity profiles in children and older subjects living in malaria-endemic zones comparatively to none-exposed healthy controls. *Methods*. Analysis of IgG self-reactive footprints was performed using Western blotting against healthy brain antigens. Plasmas of 102 malaria exposed individuals (MEIs) from endemic zone, with or without cerebral malaria (CM) were compared to plasmas from non-endemic controls (NECs). Using linear discriminant and principal component analysis, immune footprints were compared by counting the number, the presence or absence of reactive bands. We identified the most discriminant bands with respect to age and clinical status. *Results*. A higher number of bands were recognized by IgG auto-antibodies in MEI than in NEC. Characteristic changes in systemic self-IgG-reactive repertoire were found with antigenic bands that discriminate *Plasmodium falciparum* infections with or without CM according to age. 8 antigenic bands distributed in MEI compared with NEC were identified while 6 other antigenic bands were distributed within MEI according to the age and clinical status. Such distortion might be due to evolutionary processes leading to pathogenic/protective events.

## 1. Introduction

*Plasmodium falciparum* infection remains a major cause of death in most of developing countries, particularly in sub-Saharan Africa [1]. It generates different clinical manifestations with either few or no symptoms [2] or severe signs such as impaired neurological function termed cerebral malaria (CM) [3, 4]. These variable courses of the disease have been related to different factors such as the levels of parasites transmitted, the age of the infected subjects, the genetic background of the population studied [3, 4], and, above all, the immune status of affected individuals [2]. Thus, unprotected children living in endemic zones or unprotected adults coming from nonendemic zones have a high susceptibility to severe malaria [5].

It is postulated that partial protective immunity can be induced after iterative infection through triggering the action of the immune response, particularly the humoral response [6, 7]. Recent studies have highlighted the influence of the autoantibody response [8, 9]. Therefore, appropriate analysis of the serum self-IgG repertoire could contribute to a better understanding of the immuneregulation processes involved during the course of the disease [10].

In healthy subjects, despite of interindividual differences, the human serum self-IgG response is thought to be well conserved and restricted to the recognition of a few self-antigens in autologous tissues [11]. In contrast, durable distortion of these immune profiles has been found in our laboratory among patients with multiple sclerosis (MS) or other autoimmune diseases with predominant neurological signs such as neuropsychiatric systemic lupus erythematosus [12, 13]. When we induced experimental autoimmune encephalomyelitis, dynamic changes in immune profiles related to pathogenic or protective events were also identified [14, 15]. Despite the predominant neurological symptoms in clinical and experimental situations, discriminant self-IgG reactivity involves mostly ubiquitous antigens rather than specific targets in nervous system tissue [16]. Although these footprints have allowed the identification of new useful biomarkers [12, 13], their pathophysiological significance remains to be defined.

In the present study, we aimed to evaluate the impact of the environment and self-reactive natural and acquired antibody repertoires on humoral immune profiles. The findings of numerous epidemiological and clinical studies suggest that the risk of allergic and autoimmune diseases is related to the hygiene hypothesis [17]. Parasitic infections, especially malaria, may influence the development, or the course of autoimmune disease such as MS [18]. In contrast some self-reactive antibody responses might also influence the course of malaria leading to protective [8] or pathogenic events [19].

To further evaluate the relationships between environmental factors, autoimmune profiles, and the clinical status of malaria, the natural and acquired self-IgG antibody responses were analyzed in subjects of different age brackets living in endemic zones of parasitic transmission. Immune profiles were compared between malaria patients with (cerebral malaria) and without (including uncomplicated disease, asymptomatic *Plasmodium falciparum* carriers) neurological symptoms. Nonimmune individuals living in countries free of malaria were tested as controls. Our data revealed the presence of antigenic bands specifically targeted by plasma IgG collected in patients of a well-defined clinical status and age range. The pathophysiological significance of such new biomarkers is discussed.

## 2. Methods

### 2.1. Population Studied: Clinical Criteria

Plasma self-IgG reactivity against brain tissue antigens was evaluated in 119 subjects. Blood samples were collected from subjects exposed to malaria parasite, termed malaria-exposed individuals ([MEI] *n* = 102, mean age ± SD: 21.07 ± 20.2), and from healthy subjects living in European nonendemic areas, termed nonendemic controls ([NEC] *n* = 17, mean age ± SD: 39 ± 4.5).

The MEI group was divided into two subgroups. One subgroup consisted of patients with neurological symptoms, usually termed cerebral malaria ([CM] *n* = 28, mean age ± SD: 16.2 ± 21.4). The other subgroup was termed MEI without neurological symptoms (*n* = 74, mean age ± SD: 21.1 ± 21.3) and was comprised of parasite carriers with classical symptoms of malaria but without other complications and asymptomatic carriers (without any detectable symptoms). MEIs with symptoms (neurological and classical) were recruited from the Emergency Department of the University Hospital of Cocody-Abidjan (Côte d'Ivoire). Using available data bases, plasma was classified on the basis of World Health Organization (WHO) guidelines. Thus, MEI with neurological symptoms included patients with a Blantyre Coma scale ≤2 (concerning the children), a modified Glasgow coma scale ≤9 (concerning the adult), the occurrence of at least one convulsive episode during the 24 h before admission to the emergency department, and *Plasmodium falciparum*-positive thin blood smears. As expected, all of these patients were cured after quinine therapy.

Blood samples of asymptomatic parasites carriers were obtained from the National Blood Transfusion Centre of Abidjan, from the Mother-Infant Department of the District Hospital, and from some volunteer students. All subjects were included after informed consent from themselves or from their parents. All the procedures were reviewed and approved by the National Health Office Ethics Committee of Côte d'Ivoire.

### 2.2. Population Studied: Biological Criteria

For the NEC group, blood samples were obtained from healthy volunteers following approval by the “Centre de Ressources Biologiques-Centre d'Investigation Clinique” of CHRU-Lille. Isolated plasmas were then stored at −40°C in our laboratory (EA 2686, Université Lille-Nord de France).

For the MEI group, the samples were taken on the day of admission (in the emergency department before any therapy concerning those who were ill, the Mother-Infant Department, the National Centre of Blood Transfusion, or in the school for those who were asymptomatic parasite carriers). Venous blood was collected into EDTA sterile vacutainers (5 mL). Plasma was isolated after centrifugation at 500 ×g for 15 minutes and stored at −40°C at the “Laboratoire d'Immunologie of CHU Cocody-Abidjan” before being sent the EA 2686 Research Group (Lille-France).

Parasitemia was assessed at the department of “Parasitologie-Mycologie, Faculté de Médecine d'Abidjan-Cocody-Côte d'Ivoire” as follows.

Giemsa-stained thick and thin blood films were prepared for detection and identification of *Plasmodium falciparum *and for parasite counts. Parasitemia is expressed as the percentage of infected red blood cells.

Anti-falciparum total antibodies detection and quantification were performed at the “Laboratoire de Parasitologie Mycologie-Centre de Biologie, de Pathologie-CHRU de Lille-France.” Acetone-fixed *Plasmodium falciparum* strain 3D7 was used as antigen using indirect immunofluorescence. Briefly, sera were diluted 1/100, 1/200, 1/400, 1/800, 1/1600, and 1/3200 in phosphate-buffered saline (Ref 77511 Biomerieux). Diluted serum was incubated with acetone-fixed *Plasmodium falciparum* at 37°C for 30 mn. The secondary antibody used was fluorescein-conjugated goat anti-human IgG, IgM, IgA, Heavy and Light chains-H, and L-(Ref 74511 Diagnostic Pasteur) in Bleu Evans solution (Ref 75491 Biomerieux). Any fluorescence in Fluopep (Ref 75521 Biomerieux) obtained at a dilution 1/100 was considered negative. Quantification was expressed by the titre of total antifalciparum antibodies.

### 2.3. Brain Samples

Brain samples were obtained as previously described [20, 21]. Briefly, cerebral tissue was extracted by autopsy from the frontal lobe in Broadman's area 10, from healthy subjects with no history of neurological disease (Department of Neuropathology, Centre Hospitalier de l'Université de Lille, and Institut National de la Santé, de la Recherche Médicale, Unité 422, Lille, France). Autopsies were performed within the framework of a tissue collection program that had been approved by the local ethics committee.

### 2.4. Sodium Dodecyl Sulfate—Polyacrylamide Gel Electrophoresis (SDS-PAGE)

The brain samples were homogenized in a Tris buffer containing 5% SDS at a final concentration of 10 mg/mL and heated at 95°C for 10 min. 100 *μ*L of this lysate was loaded onto a 10–20% gradient polyacrylamide gel (Navix Tris-Glycine Gels-Invitrogen), beside a well of 15 *μ*L of molecular mass marker (Amersham Pharmacia Biotech). Just before 1-dimension electrophoresis (1D), the homogenates were reduced for 10 min with 10 mM DTT (Sigma-Aldrich) at 100°C. Then electrophoresis was run in Laemmli buffer using the following conditions: run time 2 h at constant 125 V and 30–40 mA/gel (start); 8–12 mA/gel (end).

### 2.5. Western-Blot Analysis

Proteins were transferred onto ECL nitrocellulose membranes (Amersham Pharmacia Biotech) at 0.8 mA/cm^2^ and later saturated with 5% fat-free dried milk. Nitrocellulose membrane was cut into 15–20 strips, 3 to 4 mm wide. Western blotting was conducted with total sera, diluted 1/100 in TNT (100 mM Tris—pH 8.3-, 0.3 M NaCl containing 0.5% Tween) and 5% fat-free dried milk. Reaction IgG antibodies were revealed with an anti-human Fc*γ* HRP-conjugated antibody (1/10000; Sigma-Aldrich) after overnight incubation at 4°C. Fluorograms were prepared using an enhanced chemiluminescence kit (Amersham Pharmacia Biotech Europe). Immune profiles were analyzed when 2 independent assays had produced identical patterns. Image analysis was performed on a Molecular Imager GS-800 Calibrated Densitometer (Bio-Rad) to localize and compare the IgG immune profile patterns. Band detection, alignment, and matching of the antibody reactivities were performed using Phoretix 1D and 1D Pro software version 10 (Totallab-Nonlinear Dynamics) and validated by two operators. We performed comparative analyses using detection-band parameters that allowed us to consider each band intensity that was >13% higher than the global background intensity of the gel to be significant. To calibrate and more accurately define the alignment of antibody reactivities, molecular weight marker protein standards (StdMW-Benchmark Pre-Stained Protein Ladder, Invitrogen) as well as internal reference standards were used. The level to which each molecular standard migrates is called relative front of migration (RF). A function curve defined by RF values on the *X* axis and standard molecular weight on the *Y* axis allowed the molecular weight of each antigenic band to be determined by extrapolation because the reactive band align on its RF.

### 2.6. Statistical Analysis

Data were expressed in binary mode (0 = absence of an antigenic band and 1 = presence of an antigenic band) for submission of IgG antibody patterns for analysis using either the chi-square test or Fisher's exact test. This approach allowed us to select the most relevant antigens that qualitatively supported different types of immune recognition. We then used linear discriminant analysis (LDA) to balance the discriminating antigens between the populations of individuals, as described previously [22, 23]. Using a stepwise logistic regression model and supported by the LDA method, we isolated a subgroup of brain antigens according to their strength of discrimination between the different populations involved in the study. Using 2 parameters (for the presence—1—or absence—0—of each selected antigen) and the coefficient previously defined by the LDA, a score was calculated for each subject as a representative value of the individual immune profile, using the following formula:

(1) $$\begin{array}{cc} \text{Score} & =\text{Ag}1\text{coef}1\times \left[0\left(\text{absent}\right)\;\text{or}\;1\;\left(\text{present}\right)\right]\\ & \;+\text{Ag}2\text{coef}2\times \left[0\left(\text{absent}\right)\;\text{or}\;1\;\left(\text{present}\right)\right]+\text{Ag}3\text{coef}3, \end{array}$$

where Ag represents antigen and coef represents coefficient.

The calculated scores were then represented graphically. A threshold value was determined using a receiver operating characteristic curve, and the sensitivity and specificity of this approach were evaluated. When the number of patients was too small to apply LDA, chi-square and Fisher's exact tests were performed to retain the most discriminant band. To compare the number of bands between the different groups of subjects, we used the chi-square test. The frequency of each discriminant self-reactive band has been calculated determining its percentage of its presence within the group of subjects.

Using principal component analysis (PCA) and nonparametric test of Kruskal-Wallis, we evaluated the relationship between the discriminant band and the clinical status.

## 3. Results

### 3.1. Comparative Analysis of Self-IgG Patterns Obtained in Subjects Living in Endemic or in Nonendemic Areas for *Plasmodium falciparum* Infection

**Figure 1:**
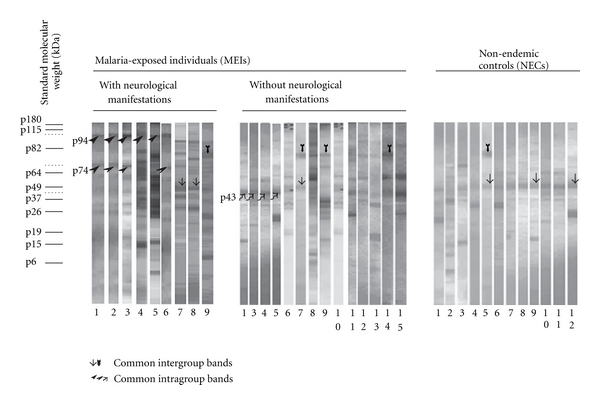
Representative IgG autoreactive patterns obtained with plasma from subjects living in malaria endemic area. The tested plasmas were obtained from malaria-exposed individuals (MEIs) with or without neurological manifestations, of all ages (from 1 to 86 years old). In parallel nonexposed healthy subjects called nonendemic controls (NECs) were also evaluated. For these western blot analyses, protein extracts of the central nervous system (CNS) tissue collected in one subject with no history of neurological disease were used as antigenic targets. Despite the heterogeneous band patterns, comparison of the strips allowed identification of some singular antigenic bands. Some bands were shared by different groups of subjects (vertical arrows). In contrast, other bands were specifically found in some groups of subjects (diagonal arrows). As illustrated, p94 and p43 protein bands were specifically found in either MEI with neurological symptoms or in MEI without neurological symptoms.

Self-IgG reactivity against human brain was compared between patterns obtained with plasma samples from the MEI group, with or without neurological symptoms (subjects from 1 to 86 years old), and those obtained with samples from the NEC group (Figure 1). Self-IgG responses appeared to be quantitatively (number of bands) and qualitatively (presence or absence of bands) heterogeneous within or between the different populations studied.

 Quantifiable data were given after the mapping and alignment of all patterns which revealed 4 to 19 bands according to the strips. When all of the strips were considered, there were more antigenic bands in the MEI (*n* = 102, mean ± SD: 14.9 ± 3.9) than in NEC group (*n* = 17, mean 4.7 ± 1.9; *P* < 0.0001). Comparison of subjects within the MEI group did not reveal any significant differences (MEI with neurological symptoms, *n* = 28, mean: 14.2 ± 3.8; MEI without neurological symptoms, *n* = 74, mean: 15.45 ± 3.9). Furthermore, no correlation was found between the number of protein bands within MEI and the titre of antifalciparum antibodies evaluated by the procedure described in materials and methods (parametric test of Pearson used for MEI with neurological symptoms, *P* = 0.625 and in MEI without neurological symptoms, *P* = 0.974).

For qualitative analysis, the presence or the absence of each protein band for all bands identified in the 119 strips (*n* = 42) was evaluated on each strip. Despite interindividual differences, some conserved sets of protein bands were found. As indicated in Figure 1, common responses were noted either in the same group (MEI with neurological manifestations) or between distinct groups (MEI with neurological symptoms, MEI without neurological symptoms, and NEC).

### 3.2. Identification of Discriminant Antigenic Bands Targeted by Self-IgG

#### 3.2.1. Related to the Clinical Status

**Figure 2:**
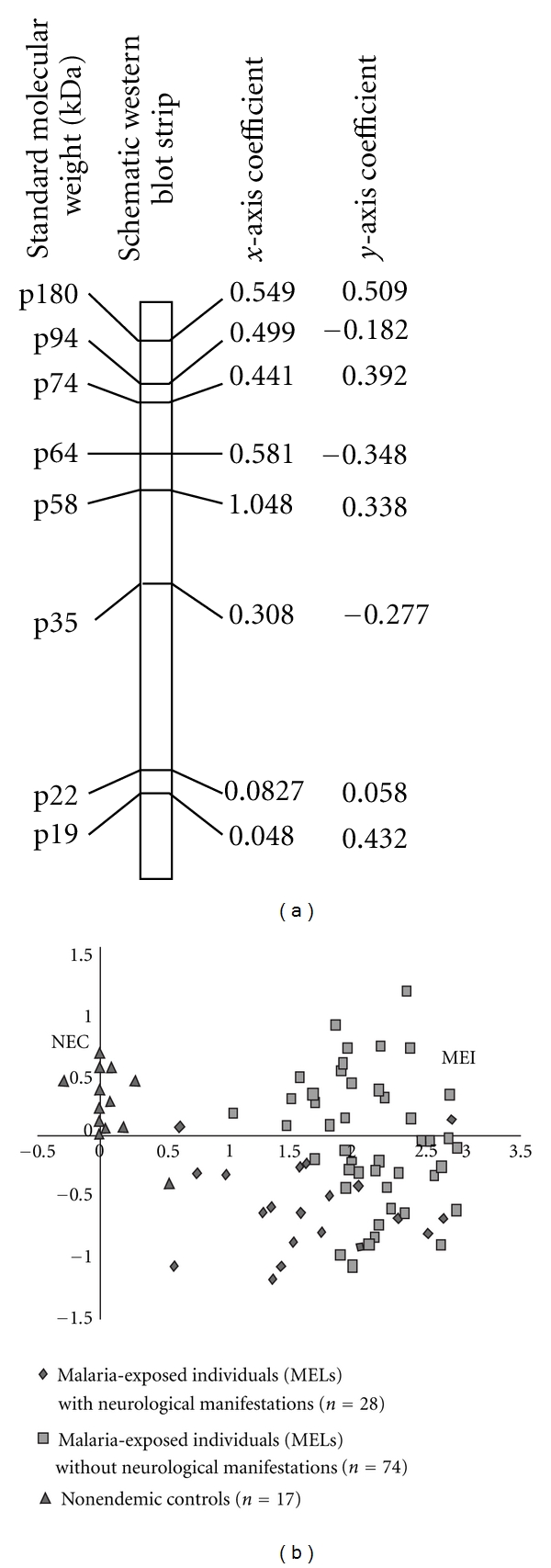
Identification of antigenic bands discriminating MEIs with or without neurological manifestations and NECs. (a) Schematic representation of 1 western-blot strip, showing the 8 healthy brain antigenic bands (characterized by their molecular weight) that reveal discriminatory immune reactivities with sera from MEI with or without neurological manifestations and NEC. The coefficient values assigned by linear discriminant analysis (LDA; coefficients for the *x* axis and *y* axis) are specified for each antigenic band. (b) Graphic extrapolation of the LDA data, obtained from the plasma tests of the 3 clinical groups of subjects revealed three distinct zones in which the NEC subjects were clearly separate from MEI.

After chi-squared and Fisher analyses, 8 antigenic bands (p180, p94, p74, p64, p58, p35, p22, and p19) showed a different distribution in MEI *versus* NEC. The coefficient value determined by LDA for each discriminatory antigenic band associated with the presence or absence of these antigens allowed us to calculate the graphic coordinates for each subjects. As shown in Figure 2, the score assigned to each subject revealed three distinctive areas according to each group. We also observed that the NEC group was largely distinct from the MEI group.

#### 3.2.2. Related to Age

Age is already known to influence the course of malaria infection [5]. We therefore considered the parameter of age with arbitrary distinction of 4 age brackets (1–5, 6–15, 16–30, and >30 years) to detect any antigenic bands that discriminate between malaria with or without neurological symptoms.

**Figure 3:**
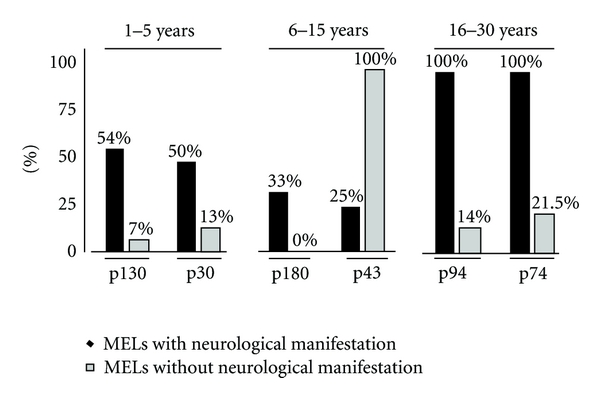
Evolution of IgG autoreactive patterns related both to age groups and clinical status within MEI. In each age group, distinct discriminant antigenic bands were found: p130 and p30 (1–5 years), p180 and p43 (6–15 years), and p94 and p74 (16–30 years). The respective frequencies of these bands were given for MEI with (black diagram) or without (grey diagram) neurological manifestations.

Chi-square, Fisher, and LDA analyses were performed to discriminate between MEI with or without neurological symptoms for each of the defined age groups. For ages over 30 years, LDA combined with Fisher and chi-square tests did not identify any discriminatory antigenic bands, in MEI with or without neurological symptoms. However, for ages under 30 years, LDA and Fisher tests identified the two most discriminant antigenic bands in MEI with or without neurological symptoms (with *P* < 0.250) per age group (1–5 years: p130 and p30; 6–15 years: p180 and p43; 16–30 years: p94 and p74). As shown in Figure 3, comparative chi-square analyses (with *P* < 0.05) of band frequencies showed no discriminatory bands to be coexpressed in two age groups. Therefore, this excludes frequency changes related to age and clinical status. Only the p94 and p74 antigenic bands (Figure 1) are expressed in 100% of MEI subjects aged to 16–30 years with neurological symptoms. Only the p43 antigenic band (Figure 1) was expressed in MEI subjects aged to 6–15 years old.

**Figure 4:**
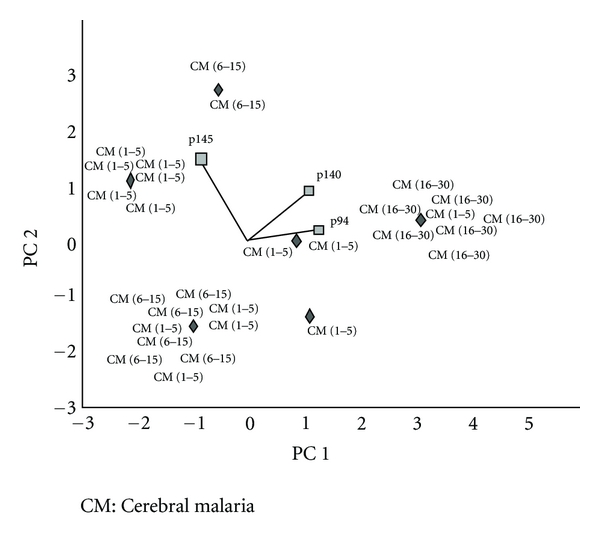
Relationship between p94 antigenic band and MEI with neurological manifestations. A two-dimensional principal component analysis (PCA) of all MEI with neurological manifestations (termed cerebral malaria-CM) aged from 1 to 30 years was evaluated. The biplot of the data (PC 1 = *x*-axis *versus* PC 2 = *y*-axis) displays the correlation between the expression of the discriminatory bands (shown as lines) and the age group of CM patients (in italics for 1–5 years; in bold for 6–15 years; in normal characters for 16–30 years). For each patient, data dispersions were represented by the barycentre (*◆*). The position of the invidious relative to the three discriminant bands (shown as lines) reveals their relationship. In this view, p94 appears to be the best marker associated with CM aged to 16–30 years.

 To confirm a tight relationship between MEI with neurological symptoms or CM, a complementary LDA was performed which accounts for the occurrence of CM irrespective of the age of MEI. Three bands including p94, p140, and p145 were then identified (data not shown) to discriminate between CM (1–5 years old), CM (6–15 years old), and CM (16–30 years old). The 2-dimensional PCA shown in Figure 4 indicates p94 as the best biomarker for a well-defined clinical status within a well-defined age range (Kruskal-Wallis test: *P* = 0.001).

## 4. Discussion

*Plasmodium falciparum *underlies most cases of severe and fatal malaria. Among the complications of this preeminent tropical parasitic disease, CM and severe anemia are the most common causes of death, especially in children under the age of five years, and in pregnant women and malaria-naïve individuals [24]. Although the risk factors that predispose individuals to develop CM are still largely unknown [25], the lack of premunition defined by the antimalarial immune response is often evoked [4]. These data underline how the capacity of the immune system plays a critical role in pathophysiological events associated with malaria. In our study, we focused on some properties of the humoral response by studying the repertoire of self-reactive IgG antibodies. Changes in the humoral response in malaria have been previously characterized by high plasma levels of IgG and IgM antibodies [26, 27] although only a small proportion of these antibodies are directed against malaria antigens [28]. In contrast, high frequencies of autoantibodies with specificities comparable with those associated to autoimmune diseases have been also described in this context [9]. The exact causes and consequences of natural and/or acquired autoimmune responses associated with malaria infections remain to be defined. In the present study, we evaluated the global self-reactive IgG antibody patterns against brain antigens obtained with plasma from 102 individuals living in high perennial transmission area (malaria exposed individuals or MEIs) with or without CM. We compared these with the IgG antibody patterns against brain antigens from the plasma samples of 17 nonexposed healthy subjects (nonendemic controls, NECs).

A comparative analysis of all patterns obtained regardless of the subjects' age (range from 1 to 86 years old) allows us to first discuss the significance of observed quantitative changes. The MEI group showed a higher number of antigenic bands detected by plasma IgG compared with the NEC group. This could be due to a higher external antigenic stimulation in MEI with a polyclonal B cell activation induced by parasites acting as mitogens [29, 30] with a potent effect on the natural and acquired antibody repertoire. Such an elevated self-IgG reactivity may be also related to the parasitic genetic diversity with a possible process of molecular mimicry [31]. Indeed, *Plasmodium falciparum* has been shown to have considerable genetic diversity, which is facilitated by the haploid state of all plasmodial stages in the human host. This genotypic variability is expressed by a microgeographical heterogeneity of the parasite [31]. In CM, the antiplasmodial antibodies induced may react directly against the numerous parasite-derived antigens and cross react against a few epitopes expressed on brain parasite-induced endothelial microparticles [32, 33]. In addition, brain endothelial-derived microparticles with the properties of cell-adhesion molecules are increased in patients with malaria [34–37] and can be also recognized as autoantigens. Thus, shared epitopes between parasite-infected erythrocyte surface molecules and self-host antigens may be targeted by antifalciparum antibodies and can lead to increased self-immune reactivity against the brain extract tissues used in our Western-blot tests. In contrast to a previous study [38], we found no correlations between the number of protein bands recognized by self-IgG antibodies to brain antigens and the titer of total antifalciparum antibodies. Specific antibodies quantified by Elisa were detected against only some representative parasitic antigens [38]. In our work, we have tested antibody response against surface antigens (data not shown). It would be of interest to further evaluate IgA and IgM self reactivities against brain antigens to estimate a potent correlation with specific response against parasite antigens. Beside the question of distinct methodological approaches (large spectrum of affinity for detected IgG in western blot, restricted high affinity for specific antibodies against *Plasmodium falciparum* antigens evaluating IgG but also IgA and IgM isotypes); such data suggest a possible involvement of natural and/or acquired autoantibody responses in the observed patterns. The participation of the natural autoantibody repertoire is also suggested by some protein bands commonly recognized by all tested plasmas (MEI and NEC groups). Previous data underlined the presence of such consensual immune patterns of self-recognition defined as immunological homunculus [39–41].

A comparative analysis of all patterns, obtained whatever the age of the subjects, allows us to also discuss the significance of observed qualitative changes. Indeed, high interindividual differences in autoreactivity are found among all tested subjects. There is an additional problem linked to the genetic control of such a response, thus, causing a huge variability in terms of population genetics. Nevertheless, in spite of such degree of heterogeneity with regard to the number and the nature of the protein bands recognized, the statistic analysis of such qualitative changes allowed us to clearly differentiate the three groups of tested subjects (MEI with or without CM and NEC) with high sensitivities and specificities. Thus, we defined 8 brain antigens differentially represented in the MEI group compared to the NEC group; and 6 other brain antigens differentially represented inside the MEI group in relation, the age (before 30 years old) and to the clinical status (with or without CM). Such distortion of the global systemic self-IgG-reactive repertoire might be caused by variable events inducing changes in natural or adaptive tolerance. Indeed, humoral innate responses with natural autoantibodies, detected in absence of known immunization [39, 40], provide immediate and broad protection against pathogens but also participate in the establishment of the natural tolerance [40]. Recent data have shown relationships between the activation of TLRs and the production of natural antibodies. Upon TLRs signals, hyper somatic mutation and class-switch recombination have been demonstrated [42]. Thus, exogenous (Plasmodium infection) but also endogenous factors (events related to malaria pathogenesis) might induce the breakdown of natural tolerance mechanisms with or without evident symptoms [14] even if it has been reported that autoimmune responses may also play a role in the protective immune process in malaria [8].

To more precisely define the factors involved in the changes, the two MEI subgroups (MEI with or without CM), we considered the parameter of age using an arbitrary distinction of 4 age brackets (1–5, 6–15, 16–30, and >30 years). Age influences the severity of the infection since it is known that malaria in children living in endemic zones is often severe. They are the first victims of CM because of their unprotected immune status [5, 43, 44]. In our study, two types of singular self-IgG reactivity were noted (common or discriminatory antigenic bands) when we examined the three youngest age groups of MEI with or without neurological signs (1–5 years, 6–15 years, and 16–30 years). The lowest number of MEI with CM was for the subgroup aged over 30 years and does not allow us to discuss the results obtained for this age group. However, all self immune prints from MEI, with or without neurological symptoms, were superimposed in this age bracket. These data on self-reactive prints suggest that during parasitic infection of adults, there is a point where stop-evolving events appear. During aging, internal changes in self-proteins may occur, and the general network of natural autoantibodies called “Immunculus” [45] will be also modified [46]. Furthermore, some internal events (metabolic deviations related to diseases) and external events (environmental factors) could lead to “immunculus” distortions. Common antigenic bands (p49) appeared in MEI age groups from 1 to 30 years irrespective of clinical status. This nondiscriminatory reactivity might be linked to the previously described “immunculus” characterized by constant or minimal individual variations of the immune repertoire [45]. These natural autoantibodies derived from human germline immunoglobulin genes without somatic mutations are often poly reactive and expressed a low affinity for antigens [39, 40, 47]. Natural autoantibodies in different members of a malaria population may also react against the conserved public self-epitopes [9, 48–50]. In return, the microbial, viral, and parasitic infections can all modify the antibody repertoire leading to the emergence of potent pathogenic auto-antibodies [51]. Natural and acquired auto-antibodies are often directed against ubiquitous antigenic targets with different seric titers, that is, antinuclear, antimuscle, or antiphospholipid, antibodies. Nevertheless, they occur at higher frequencies among individuals exposed to viral, bacterial, and parasitic diseases, including malaria [9, 40].

In our work, some specific antigenic bands, presented as a peculiar intragroup self-reactivity pattern, are expressed according to each age group in different proportions in MEI with or without CM. In children aged 1 to 15 years, discriminant antigenic bands were identified (p130-p30 in subjects aged from 1 to 5 years, and p180-p43 in subjects aged from 6 to 15 years). However, they are not good bio-markers for CM, as they were low in frequency within each age group of MEI with CM. In contrast, in MEI subjects aged from 16 to 30 years, significant discriminatory bands were found. Subjects of this group displayed singular self-immune reactivity—characterized by only two discriminatory self-reactive antigenic bands—p94 and p74—when the presence or absence of CM is taken into account. This appears stable in all subjects since they were immunized against almost all prevalent parasitic strains encountered in the area [4, 52, 53]. When we considered only the presence of CM irrespective of age, three antigenic bands were identified (p145, p140, and p94). A double band localized at p147 KDa has been previously detected and characterized as isoforms of nonerythroid spectrin [38]. Regarding the degree of resolution of our methodological approach of we cannot exclude that p140, p145, and p147 could be the similar antigenic targets. The p94 band is of particular interest. It discriminated, not only MEI and NEC, but also it discriminated both MEI with or without neurological symptoms and CM patients according to age. As previously postulated, this new discriminant antigenic target could be a fraction of the p147 band, cleaved during apoptosis following the activation of a neutral calcium-activated protease (calpain) [54]. Such dynamic changes occurring before and after 16 years might be related to the immune response against different strains of *Plasmodium falciparum* that underlie recurrent infections. Indeed, in malaria-endemic zones, acquired protective immunity (called premunition) induced by parasitic diversity is strengthened with increasing age [55–57]. Consequently, in children, iterative infection episodes may induce antibodies to specific parasitic strains which can cross-react against self-epitopes. Furthermore, these recurrent parasitic attacks occurring in childhood may induce inflammatory and deleterious tissue events. This may generate neoantigenicity by epitope spreading processes [58], or by revealing self cryptic/hidden antigens [14, 15, 59], thereby, favoring expression of new antibodies involved in self-immune reactivity and potent pathophysiological events.

## 5. Conclusion

The present work is focused on the age-related evolution of the self-immune patterns. Therefore, the characterization of the nature and structure of identified antigenic bands of interest (such as p94) will be assessed in future studies requiring mass-spectrometry analysis. Their presence in malaria infection may be of prognostic interest as in other infections where some auto-antibodies reveal a new repertoire that heralds susceptibility to future autoimmune diseases [60]. Self-reactivity to nonerythroid alpha spectrin and beta tubulin III have been associated with CM in Gabonese children [38] and Indian populations, respectively [61]. However, to our knowledge, no specific brain auto-antigens related to age of infection with malaria have been reported until now. It is, therefore, of interest to characterize such self-antigens because CM is one of the principal causes of neurodisability in sub-Saharan Africa [62]. Although a few studies suggest that there is full neurological recovery in CM [35, 63, 64], it has become clear that many children have sustained severe brain injury over the past 15 years. Thus, 25% of CM cases result in long-term neurological and cognitive deficiency or epilepsy [65, 66]. Our data show that parasite pressure seems to cause quantitative and qualitative changes of systemic self-IgG immune-reactive profiles in MEI. However, the singularities of such an immune response related to age allow the identification of some specific antigenic bands as potent useful biomarkers. Their further characterization is of particular prognostic interest in CM since such evolutionary changes involving the repertoires of self-natural antibodies and acquired antibodies may lead to pathogenic or protective events [15].

## References

[1] Breman JG, Egan A, Keusch GT (2001). Introduction and summary: the intolerable burden of malaria: a new look at the numbers. *American Journal of Tropical Medicine and Hygiene*.

[2] Pérignon JL, Druilhe P (1994). Immune mechanisms underlying the premunition against *Plasmodium falciparum* malaria. *Memorias do Instituto Oswaldo Cruz*.

[3] Mazier D, Nitcheu J, Idrissa-Boubou M (2000). Cerebral malaria and immunogenetics. *Parasite Immunology*.

[4] Shigidi MMT, Hashim RA, Idris MNA, Mukhtar MM, Sokrab TEO (2004). Parasite diversity in adult patients with cerebral malaria: a hospital-based, case-control study. *American Journal of Tropical Medicine and Hygiene*.

[5] Yazdani SS, Mukherjee P, Chauhan VS, Chitnis CE (2006). Immune responses to asexual blood-stages of malaria parasites. *Current Molecular Medicine*.

[6] Bouharoun-Tayoun H, Attanath P, Sabchareon A, Chongsuphajaisiddhi T, Druilhe P (1990). Antibodies that protect humans against *Plasmodium falciparum* blood stages do not on their own inhibit parasite growth and invasion in vitro, but act in cooperation with monocytes. *Journal of Experimental Medicine*.

[7] Cohen S, McGregor IA, Carrington S (1961). Gamma-globulin and acquired immunity to human malaria. *Nature*.

[8] Zanini GM, de Moura Carvalho LJ, Brahimi K (2009). Sera of patients with systemic lupus erythematosus react with plasmodial antigens and can inhibit the in vitro growth of *Plasmodium falciparum*. *Autoimmunity*.

[9] Daniel-Ribeiro CT, Zanini G (2000). Autoimmunity and malaria: what are they doing together?. *Acta Tropica*.

[10] Stahl D, Lacroix-Desmazes S, Mouthon L, Kaveri SV, Kazatchkine MD (2000). Analysis of human self-reactive antibody repertoires by quantitative immunoblotting. *Journal of Immunological Methods*.

[11] Mouthon L, Haury M, Lacroix-Desmazes S, Barreau C, Coutinho A, Kazatchkine MD (1995). Analysis of the normal human IgG antibody repertoire: evidence that IgG autoantibodies of healthy adults recognize a limited and conserved set of protein antigens in homologous tissues. *Journal of Immunology*.

[12] Lefranc D, Almeras L, Dubucquoi S, de Seze J, Vermersch P, Prin L (2004). Distortion of the Self-Reactive IgG Antibody Repertoire in Multiple Sclerosis as a New Diagnostic Tool. *Journal of Immunology*.

[13] Lefranc D, Launay D, Dubucquoi S (2007). Characterization of discriminant human brain antigenic targets in neuropsychiatric systemic lupus erythematosus using an immunoproteomic approach. *Arthritis and Rheumatism*.

[14] Zephir H, Almeras L, El Behi M (2006). Diversified serum IgG response involving non-myelin CNS proteins during experimental autoimmune encephalomyelitis. *Journal of Neuroimmunology*.

[15] El Behi M, Zéphir H, Lefranc D (2007). Changes in self-reactive IgG antibody repertoire after treatment of experimental autoimmune encephalomyelitis with anti-allergic drugs. *Journal of Neuroimmunology*.

[16] Almeras L, Lefranc D, Drobecq H (2004). New antigenic candidates in multiple sclerosis: identification by serological proteome analysis. *Proteomics*.

[17] Bach JF (2002). The effect of infections on susceptibility to autoimmune and allergic diseases. *New England Journal of Medicine*.

[18] Sotgiu S, Angius A, Embry A, Rosati G, Musumeci S (2008). Hygiene hypothesis: innate immunity, malaria and multiple sclerosis. *Medical Hypotheses*.

[19] Sorensen PG, Mickley H, Schmidt KG (1984). Malaria-induced immune thrombocytopenia. *Vox Sanguinis*.

[20] Archelos JJ, Storch MK, Hartung HP (2000). The role of B cells and auto antibodies in multiple sclerosis. *Annals of Neurology*.

[21] Diamant M, Tushuizen ME, Sturk A, Nieuwland R (2004). Cellular microparticles: new players in the field of vascular disease?. *European Journal of Clinical Investigation*.

[22] Pugliatti M, Sotgiu S, Rosati G (2002). The worldwide prevalence of multiple sclerosis. *Clinical Neurology and Neurosurgery*.

[23] Poser CM, Paty DW, Scheinberg L (1983). New diagnostic criteria for multiple sclerosis: guidelines for research protocols. *Annals of Neurology*.

[24] World Health Organization (1998). Malaria Fact Sheet. *World Health Organization Fact Sheet*.

[25] Medana IM, Chaudhri G, Chan-Ling T, Hunt NH (2001). Central nervous system in cerebral malaria: “Innocent bystander” or active participant in the induction of immunopathology?. *Immunology and Cell Biology*.

[26] Rowe DS, McGregor IA, Smith SJ, Hall P, Williams K (1968). Plasma immunoglobulin concentrations in a West African (Gambian) community and in a group of healthy British adults. *Clinical and Experimental Immunology*.

[27] Turner MW, Voller A (1966). Studies on immunoglobulins of Nigerians. I. The immunoglobulin levels of a Nigerian population. *Journal of Tropical Medicine and Hygiene*.

[28] Curtain CC, Kidson C, Champness DL, Gorman JG (1964). Malaria antibody content of gamma2–7S globulin in tropical populations. *Nature*.

[29] Donati D, Mok B, Chêne A (2006). Increased B cell survival and preferential activation of the memory compartment by a malaria polyclonal B cell activator. *Journal of Immunology*.

[30] Greenwood BM (1974). Possible role of a B cell mitogen in hypergammaglobulinaemia in malaria and trypanosomiasis. *Lancet*.

[31] Miller LH, Baruch DI, Marsh K, Doumbo OK (2002). The pathogenic basis of malaria. *Nature*.

[32] van der Heyde HC, Nolan J, Combes V, Gramaglia I, Grau GE (2006). A unified hypothesis for the genesis of cerebral malaria: sequestration, inflammation and hemostasis leading to microcirculatory dysfunction. *Trends in Parasitology*.

[33] Francis L, Perl A (2010). Infection in systemic lupus erythematosus: friend or foe?. *International Journal of Clinical Rheumatology*.

[34] Doeuvre L, Plawinski L, Toti F, Anglés-Cano E (2009). Cell-derived microparticles: a new challenge in neuroscience. *Journal of Neurochemistry*.

[35] van der Wal G, Verhagen WIM, Dofferhoff ASM (2005). Neurological complications following *Plasmodium falciparum* infection. *Netherlands Journal of Medicine*.

[36] Combes V, Coltel N, Alibert M (2005). ABCA1 gene deletion protects against cerebral malaria: potential pathogenic role of microparticles in neuropathology. *American Journal of Pathology*.

[37] Fernandez V, Treutiger CJ, Nash GB, Wahlgren M (1998). Multiple adhesive phenotypes linked to rosetting binding of erythrocytes in *Plasmodium falciparum* malaria. *Infection and Immunity*.

[38] Guiyedi V, Chanseaud Y, Fesel C (2007). Self-reactivities to the non-erythroid alpha spectrin correlate with cerebral malaria in gabonese children. *PLoS ONE*.

[39] Cohen IR, Norins LC (1966). Natural human antibodies to gram-negative bacteria: immunoglobulins G, A, and M. *Science*.

[40] Coutinho A, Kazatchkine MD, Avrameas S (1995). Natural autoantibodies. *Current Opinion in Immunology*.

[41] Merbl Y, Zucker-Toledano M, Quintana FJ, Cohen IR (2007). Newborn humans manifest autoantibodies to defined self molecules detected by antigen microarray informatics. *Journal of Clinical Investigation*.

[42] Capolunghi F, Cascioli S, Giorda E (2008). CpG drives human transitional B cells to terminal differentiation and production of natural antibodies. *Journal of Immunology*.

[43] John CC, Bangirana P, Byarugaba J (2008). Cerebral malaria in children is associated with long-term cognitive impairment. *Pediatrics*.

[44] Okiro EA, Al-Taiar A, Reyburn H, Idro R, Berkley JA, Snow RW (2009). Age patterns of severe paediatric malaria and their relationship to *Plasmodium falciparum* transmission intensity. *Malaria Journal*.

[45] Poletaev A, Osipenko L (2003). General network of natural autoantibodies as immunological homunculus (Immunculus). *Autoimmunity Reviews*.

[46] Kay M (2005). Immunoregulation of cellular life span. *Annals of the New York Academy of Sciences*.

[47] Warrington AE, Rodriguez M (2010). Method of identifying natural antibodies for remyelination. *Journal of Clinical Immunology*.

[48] Jhaveri KN, Ghosh K, Mohanty D (1997). Autoantibodies, immunoglobulins, complement and circulating immune complexes in acute malaria. *National Medical Journal of India*.

[49] Anaya JM, Correa PA, Mantilla RD, Jimenez F, Kuffner T, McNicholl JM (2001). Rheumatoid arthritis in African Colombians from Quibdo. *Seminars in Arthritis and Rheumatism*.

[50] Oppezzo P, Dighiero G (2003). Autoantibodies, tolerance and autoimmunity. *Pathologie Biologie*.

[51] Moynier M, Abderrazik M, Rucheton M, Combe B, Sany J, Brochier J (1991). The B cell repertoire in rheumatoid arthritis. I. Frequency of EBV-inducible circulating precursors producing autoantibodies. *Journal of Autoimmunity*.

[52] Mendis KN, Carter R (1995). Clinical disease and pathogenesis in malaria. *Parasitology Today*.

[53] Branch OH, Takala S, Kariuki S (2001). *Plasmodium falciparum* genotypes, low complexity of infection, and resistance to subsequent malaria in participants in the Asembo Bay Cohort Project. *Infection and Immunity*.

[54] Wang KKW (2000). Calpain and caspase: can you tell the difference?. *Trends in Neurosciences*.

[55] Smith T, Felger I, Tanner M, Beck HP (1999). Premunition in *Plasmodium falciparum* infection: insights from the epidemiology of multiple infections. *Transactions of the Royal Society of Tropical Medicine and Hygiene*.

[56] Soe-Soe, Khin-Saw-Aye, Htay-Aung (2001). Premunition against *Plasmodium falciparum* in a malaria hyperendemic village in Myanmar. *Transactions of the Royal Society of Tropical Medicine and Hygiene*.

[57] Ofosu-Okyere A, Mackinnon MJ, Sowa MPK (2001). Novel *Plasmodium falciparum* clones and rising clone multiplicities are associated with the increase in malaria morbidity in Ghanaian children during the transition into the high transmission season. *Parasitology*.

[58] Yasawardene SG, Lomonossoff GP, Ramasamy R (2003). Expression & immunogenicity of malaria merozoite peptides displayed on the small coat protein of chimaeric cowpea mosaic virus. *Indian Journal of Medical Research*.

[59] Henderson KA, Streltsov VA, Coley AM (2007). Structure of an IgNAR-AMA1 complex: targeting a conserved hydrophobic cleft broadens malarial strain recognition. *Structure*.

[60] Quintana FJ, Hagedorn PH, Elizur G, Merbl Y, Domany E, Cohen IR (2004). Functional immunomics: microarray analysis of IgG autoantibody repertoires predicts the future response of mice to indunced diabetes. *Proceedings of the National Academy of Sciences of the United States of America*.

[61] Bansal D, Herbert F, Lim P (2009). IgG autoantibody to brain beta tubulin III associated with cytokine cluster-II discriminate cerebral malaria in central India. *PLoS ONE*.

[62] Birbeck GL, Taylor TE (2005). Severe malaria: still counting the costs. *Journal of Neurology, Neurosurgery and Psychiatry*.

[63] Muntendam AH, Jaffar S, Bleichrodt N, van Hensbroek MB (1996). Absence of neuropsychological sequelae following cerebral malaria in Gambian children. *Transactions of the Royal Society of Tropical Medicine and Hygiene*.

[64] Idro R, Kakooza-Mwesige A, Balyejjussa S (2010). Severe neurological sequelae and behaviour problems after cerebral malaria in Ugandan children. *BMC Research Notes*.

[65] Carter JA, Lees JA, Gona JK (2006). Severe falciparum malaria and acquired childhood language disorder. *Developmental Medicine and Child Neurology*.

[66] Carter JA, Ross AJ, Neville BGR (2005). Developmental impairments following severe falciparum malaria in children. *Tropical Medicine and International Health*.

